# Examining the Relationships Between Sleep Physiology and the Gut Microbiome in Preclinical and Translational Research: Protocol for a Scoping Review

**DOI:** 10.2196/38605

**Published:** 2022-06-21

**Authors:** Katherine Anne Maki, Jenna Alkhatib, Gisela Butera, Gwenyth Reid Wallen

**Affiliations:** 1 Translational Biobehavioral and Health Disparities Branch National Institutes of Health Clinical Center Bethesda, MD United States; 2 Division of Library Services National Institutes of Health Bethesda, MD United States

**Keywords:** microbiome, microbiota, bioinformatics, scoping review, genomics, sleep quality, sleep time, disrupted sleep, sleep, review, microbe, microbiology, search strategy, information science, library science, gut-brain, gastrointestinal, medical librarian, health science librarian

## Abstract

**Background:**

Sleep is an instrumental behavioral state with evidence supporting its active role in brain function, metabolism, immune function, and cardiovascular systems. Research supports that there are pathways underlying the bidirectional communication between the brain and gastrointestinal system, also known as the “gut-brain axis.” Primary research examining sleep and gut microbiome relationships continues to increase. Although current data include both preclinical and clinical research, gut microbiome results are reported through a wide range of metrics (alpha diversity, beta diversity, and bacterial compositional changes), which makes cross-study comparison challenging. Therefore, a synthesis of the research examining sleep and gut microbiome relationships is necessary to understand the state of the science and address gaps in the literature for future research.

**Objective:**

In this paper, we outline a scoping review protocol to evaluate and synthesize preclinical and clinical primary research focused on the associations between sleep and the gut microbiome.

**Methods:**

The search strategy was facilitated through a medical research librarian and involved electronic databases including PubMed/MEDLINE, Embase, Scopus, Web of Science, CENTRAL trials database, BIOSIS Citation Index, and the Zoological Record. Gray literature sources including medRxiv and bioRxiv preprint servers were also searched. Studies were screened according to the aims and exclusion and inclusion criteria of the protocol. After screening, data will be extracted and synthesized from the included studies according to predefined sleep and microbiome methodology metrics.

**Results:**

The search strategy yielded 4622 references that were imported for study screening, and source screening was completed in May 2022 by 2 independent investigators, resulting in a total of 93 sources for data extraction and synthesis. The data synthesis table is expected to be completed by August 2022, and the results will be disseminated through paper submission by December 2022 and presented at conferences related to neuroscience, sleep physiology, bioinformatics, and the microbiome.

**Conclusions:**

A scoping review of preclinical and clinical research is needed to synthesize the growing data focused on the relationships between sleep and the gut microbiome. We expect the results of this synthesis will identify gaps in the literature and highlight pathways linking the gut-brain axis and sleep physiology to stimulate future research questions.

**Trial Registration:**

Open Science Framework 69TBR; https://osf.io/69tbr

**International Registered Report Identifier (IRRID):**

PRR1-10.2196/38605

## Introduction

### Definition and Importance of Sleep

Sleep is defined as a reversible behavioral state associated with unresponsiveness to the external environment and involves a complex orchestration of physiological processes for its initiation and maintenance [1]. The importance of sleep is elucidated through evidence supporting its active role in several different physiological functions necessary for survival, namely immune health [2], physical growth [3], metabolism [4], tissue repair [5], and the maintenance of cognitive performance [6,7]. Sleep also contributes to the maintenance of major biological processes; in particular, it can aid in the peripheral and neurologic clearance of metabolic end products, consolidation of long-term memories, learning through long-term potentiation, and restoration of biological systems [8-10].The onset of sleep is partly controlled by circadian factors as well as homeostatic factors (ie, sleep pressure), and the interplay between circadian and sleep pressure is often referred to as the two-process system of sleep regulation [11]. In addition, intrinsic and extrinsic variables influence sleep, including the environment, work schedules, food and drink consumption, and health behaviors.

### Sleep Physiology and Pathophysiology

Reduction in sleep duration or quality (ie, sleep fragmentation) is associated with sleep disorders such as insomnia and obstructive sleep apnea [12,13] and may result from occupational factors that alter sleep patterns or habits [14]. Reduced sleep duration or quality can also be a consequence of other chronic diseases such as congestive heart failure or chronic obstructive pulmonary disease [15,16]. The reduction in substantial sleep duration or sleep quality can further contribute to increased risk of human diseases and disorders, including cardiovascular disease [17], obesity [18], and diabetes [19]. More recently, the role of the glymphatic system in waste byproduct clearance and metabolite distribution within brain tissue has been uncovered [20]. As the glymphatic system is most active during slow wave sleep [8,21], the neurologic clearance of toxins and potentially harmful byproducts is also dependent on healthy sleep patterns [22]. The association of the proper function of the glymphatic system with the prevention of cerebral amyloid angiopathy and Alzheimer disease highlights the importance of the role that adequate sleep also plays in neurologic disease prevention [23]. Molecular clocks have evolved in most living organisms to synchronize vital physiologic activities with the external environment and rotation of the Earth’s axis around the sun. The superchiasmatic nucleus is responsible for the central maintenance of the rhythm of approximately 24-hour cycles (or the circadian rhythm), is located in the hypothalamus [24,25], and responds primarily to light and darkness cues [26]. Conversely, peripheral oscillators located in many organ systems respond to other cues such as food intake, exercise, and temperature [27,28].

In mammals including humans and rodents, the oscillatory central and peripheral rhythms synchronize important functions ranging from intracellular signaling to food metabolism and immune system activity [29]. These carefully orchestrated processes are disrupted when there is a misalignment between the activities of the central and peripheral oscillators and the external environment, which may happen when traveling across time zones, alternating sleep patterns related to occupational factors such as shift work, or when sleep is disrupted due to sleep pathology (ie, insomnia or obstructive sleep apnea) [30,31]. Although altered sleep quality and duration have been linked to chronic disease risk and mortality in numerous epidemiological studies and systematic reviews [32-34], current treatment options for inadequate sleep or sleep disorders are limited. Therefore, we are also including preclinical studies in this scoping review to understand the mechanisms associated with disrupted sleep that can be expanded on and confirmed in future translational clinical research. Continued research aimed at uncovering these mechanisms of healthy and pathologic sleep has the potential to elucidate specific biomarkers and signaling mechanisms that act as mediators between sleep impairment and broader physiological changes. An initiative toward understanding and mitigating the effects of chronic sleep loss is imperative to reducing the prevalence of comorbidities and the need for health care services and its accompanying costs that otherwise may not be needed in a time of limited health care delivery resources and access.

### Gut Microbiome

The gut microbiota, or the community of bacteria in the gastrointestinal system, has been shown to display diurnal fluctuations in composition and function, which are both coordinated with host feeding time, the anticipation of nutrient digestion, and energy metabolism [35]. The human gut microbiome is characterized by the bacteria that occupy the digestive tracts of humans in combination with their collective genetic information [36]. Gut microbiome bacteria perform vital physiological functions related to nutrient intake, such as the production of metabolites and synthesizing compounds that have the potential to influence human health and pathophysiology (ie, short-chain fatty acids and trimethylamine N-oxide) [37,38]. The bacteria of the gut microbiome communicate with the brain via metabolites, immune cells, and the vagus nerve [39]. This communication, or gut-brain axis signaling, is associated with the modulation of systemic and gastrointestinal motor, sensory, and secretory functions [40]. Different physiologic and pathophysiologic states have been found to shape the gut microbiome’s bacterial content, diversity, and activity. For example, changes in the bacterial composition of the gut microbiome have been associated with diet [41], stress [39], and the external environment [42]. Due to the fact that gut microbes are associated with health maintenance through their metabolic activity and gut-brain communication, a substantial increase or decrease in specific taxa may have functional and physiologic implications for the host [43].

Pathologic gut microbiome bacterial community alterations have been linked to similar disorders associated with inadequate sleep in preclinical and translational research models, including cardiovascular disease [44], obesity [45], diabetes mellitus [46], and hypertension [47]. Furthermore, established signaling mechanisms between the gut microbiome and brain through the gut-brain axis provide the mechanistic rationale that sleep and microbiome alterations may be linked [39]. Reduced sleep duration–associated gut microbiome changes have also been associated with putative inflammatory metabolite implications such as reduced short-chain fatty acid and increased secondary bile acid levels [47,48]. Recent studies modeling sleep fragmentation and deprivation in mice have shown significant changes in the diversity of and specific bacterial taxa [47,49,50], whereas other studies found no major effects on bacterial composition [51]. Studies examining the relationship between sleep and the gut microbiome have used various models of pathologic sleep, including circadian disruption [35], sleep fragmentation [47,52,53], and reduction in sleep quantity [54] and quality [49] (Table 1).

Despite the growing research on this topic, studies focused on sleep and microbiome associations include varying sleep intervention models and intervention durations. Furthermore, a wide range of global, taxonomic, and functional microbiome analysis techniques and a lack of reporting standardization across studies create challenges in comparing the global and specific gut microbiome community responses to healthy and disordered sleep. Although the associations between sleep and gut microbiome community changes have been established, it is unknown if altered sleep influences the microbiome, the microbiome influences sleep, or sleep and the microbiome mutually modulate each other and are altered by a disruption in either system. It is also unclear if the risk for health conditions with shared associations to sleep and microbiome changes are primarily driven by pathologic changes in one or both systems, and several studies on this topic are exploratory and observational. A concern that preclinical research results may not always be replicable in human studies necessitates our synthesis of both preclinical and translational studies to evaluate congruence and disagreement across sleep and microbiome associations. Therefore, a scoping review with rigorous data abstraction, charting, and outcome measure synthesis is essential to understand gut microbiome responses across various sleep conditions in preclinical and translational clinical research.

**Table 1:** Operational definitions.

Term			Definition
**Preclinical sleep disruption grouping**			
	Mechanical sleep disruption	A form of sleep disruption that uses a machine to wake the subjects. Example: Mechanical sweeping bars across the bottom of rodent cages programmed to disrupt sleep for a specified duration [47,53]	
	Paradoxical (REM^a^) sleep disruption	A form of sleep disruption that takes advantage of muscle atonia during REM sleep to wake the subjects. Example: Platform water bath [49]	
	Circadian light alteration intervention	A form of sleep disruption that changes the light and dark cycle in the recording room (typical conditions are lights on for 12 hours/day and lights off for 12 hours/day). Examples: Constant light conditions [55] Constant dark conditions [56] Light-dark cycle inversion/switch in the recording room throughout the study protocol [57,58]	
	Biological circadian disruption	A form of sleep disruption that changes the host circadian clock through the knockout of a related gene. Examples: BMAL^b^ knockout [59] Per1/2^c^ knockout [60] Gcg-Arntl^d^ knockout [61] NPAS2^e^ (also called MOP4^f^) knockout [62]	
**Translational** **clinical sleep disruption grouping**			
	Sleep disruption	An experimental procedure used with individuals (and experimental animals) to induce partial sleep loss during their usual sleep period. Examples: Sleep deprivation [63] Sleep restriction [51]	
	Sleep pathology (with subjective and objective sleep measures)	Disorder based on poor sleep quality and sleep complaints. Examples: Insomnia [64,65] Narcolepsy [66] Obstructive sleep apnea [67] See examples of subjective and objective sleep measures below.	
**Subjective and objective sleep measures**			
	Subjective sleep measures	Pittsburg sleep quality index, subjective sleep time (hours), or time in bed or awake	
	Objective sleep measures	Actigraphy: time in bed, sleep duration, and wake after sleep onset	
**Microbiome sequencing methodology**			
	16S rRNA^g^ gene amplicon sequencing	A sequencing method that uses a variable region of the 16S rRNA gene to identify the bacteria or fungi in a given sample.	
	Shotgun metagenomics sequencing	A sequencing method that sequences all given genomic DNA from a sample. Usually has a higher taxonomic resolution than 16S rRNA sequencing and can be used for functional profiling.	
**Microbiome and microbiome-associated data items (synthesis table columns)**			
	Microbiome sample	Sample material and site (ie, proximal colon, distal ileum, fecal pellet, whole stool sample, and rectal swab)	
	DNA extraction and sequencing methodology	DNA extraction kit and protocol, the sequencing platform (ie, Illumina or Ion Torrent) and methodology used to generate microbiome sequencing reads (ie, 16S rRNA or shotgun metagenomics sequencing), and the hypervariable region of the 16S rRNA gene (if applicable)	
	Microbiome analysis considerations	Bioinformatic pipeline used to analyze microbiome data, reference database used for taxonomy identification, and statistical methods used for microbiome analysis.	
	Alpha diversity [68]	An estimate of a bacterial sample’s richness, evenness, or both. Estimators include: Shannon index Chao1 Simpson index Phylogenetic diversity	
	Beta diversity	An estimate for how much a bacterial sample differs from another. Estimators include: UniFrac metrics Bray-Curtis dissimilarity Visualization through PCoA^h^ plots in a 2D or 3D manner	
	Global phylum changes	Broad changes at the phylum level happening within a bacterial community. Generally described through the Firmicutes/Bacteroidetes ratio.	
	Differential abundance [69]	A measure of differences in the relative abundance of an individual bacteria for the purposes of comparing across conditions (ie, intervention vs control group or within group across time). Analysis methods include: ANCOM^i^ ALDEx2^j^ DESeq2^k^ EdgeR^l^ LEfSe^m^	
	Functional gene prediction	The use of bacterial genetic data (16S rRNA amplicon sequencing or shotgun metagenomics sequencing output) to gain insight on the functional potential of a bacterial community. One method is using PICRUSt2^n^ software to compare against the KEGG^o^ database.	
	Metabolomics	The study of the type and concentration of metabolites present within a given sample, which can give insight into the biochemical processes happening. Differential abundance analyses can be used to compare metabolite differences across groups, similar to microbial differential abundance.	
	Cytokine and immune markers	Types of cells that can be associated with sleep physiology changes. For example, individuals with poorer sleep may have increased levels of systemic inflammation markers, such as CRP^p^, IL-6^q^, and fibrinogen [70,71].	

^a^REM: rapid eye movement.

^b^BMAL: brain and muscle aryl hydrocarbon receptor nuclear translocator-like.

^c^Per: period.

^d^Gcg-Arntl: Gcg-Aryl hydrocarbon receptor nuclear translocator-like protein.

^e^NPAS2: neuronal PAS domain protein 2

^f^MOP4: member of PAS protein 4.

^g^16S rRNA: 16S ribosomal ribonucleic acid.

^h^PCoA: principal coordinates analysis.

^i^ANCOM: Analysis of Compositions of Microbiomes.

^j^ALDEx2: Analysis of Variance-Like Differential Expression version 2.

^k^DESeq2: Differential Expression Sequence Count Data 2

^l^EdgeR: Empirical Analysis of Digital Gene Expression in R.

^m^LefSe: Linear Discriminant Analysis Effect Size.

^n^PICRUSt2: Phylogenetic Investigation of Communities by Reconstruction of Unobserved States.

^o^KEGG: Kyoto Encyclopedia of Genes and Genomes.

^p^CRP: c-reactive protein.

^q^IL-6: interleukin-6.

### Objectives

Our objective is to explore the influence of sleep and sleep pathology on the gut microbiome in preclinical and translational clinical studies to determine the extent sleep and gut microbiome–focused research has been undertaken, what sleep disorders have been studied, the methodologies used, and whether these studies are sufficient in characterizing the global and specific bacterial gut microbiome changes (and their byproducts) associated with healthy sleep and alterations in sleep physiology. Subresearch objectives with relevant examples are presented in Textbox 1, and the operational definitions used to formulate these research questions are listed in Table 1.

**Research objectives.**

**Overarching objective**

- Explore the influence of sleep and sleep pathology on the bacterial gut microbiome in preclinical and translational research studies to determine the extent sleep and gut microbiome–focused research has been undertaken, what sleep disorders have been studied, the methodologies used, and whether they are sufficient in characterizing the global and specific bacterial gut microbiome changes (and their byproducts) associated with healthy sleep and alterations in sleep physiology.

**Subresearch objectives**

- Evaluate the extent sleep and gut microbiome–focused research has been undertaken
  Proposed action: Identify and compile all references and source material focused on the associations between sleep and the gut microbiome.
- Present which sleep disorders have been studied in the context of the microbiome
  Proposed action: Group sleep disorder, sleep disruption interventions, and the metrics of sleep into the operational definitions (see Table 1). For each group, evaluate which instruments and metrics have been reported.
- Analyze the research methodologies used and whether they are sufficient in characterizing sleep and gut microbiome community changes
  Proposed action: Evaluate which instruments and metrics are used to quantify sleep quality, quantity, and characteristics. Similarly, organize and compare the analysis strategies and measures used in the characterization of the bacterial and functional (metabolic genes and metabolites) gut microbiome community structure.
- Present the global and specific bacterial gut microbiome changes (and their byproducts) associated with healthy sleep and alterations in sleep physiology
  Proposed action: Synthesize the preclinical and translational research evaluating the associations between sleep and the gut microbiome, and systematically present the results to facilitate comparison and interpretation across studies.

## Methods

### Protocol

A scoping review of the currently published literature was chosen due to the variability of the microbiome and sleep measures reported to synthesize human and preclinical data on the associations between sleep and the gut microbiome. The PRISMA-ScR (Preferred Reporting Items for Systematic Reviews and Meta-analyses Extension for Scoping Reviews) and PRISMA-P (Preferred Reporting Items for Systematic Review and Meta-Analysis Protocols) guidelines will be used to guide the search strategy, study selection, and synthesis of results [72,73]. Briefly, PRISMA-ScR and PRISMA-P involve identifying research questions and objectives, identifying relevant studies through an electronic search strategy, study selection, and data charting and synthesis of results. The protocol for this scoping review is registered on Open Science Framework (69TBR).

### Eligibility Criteria

#### Inclusion Criteria

Primary research papers published in peer-reviewed journals will be included. Additionally, papers housed on preprint servers and conference proceedings or abstracts that adequately address the overarching research question will be included in this study. If a preprint paper or conference abstract is included in the final scoping review, a notation will be made in the summary of the included evidence table that the paper has not been peer reviewed. All study designs will be included in this scoping review, including studies with adults aged ≥18 years (human studies) and rodents (preclinical research). No date limits will be set in the search strategy, and studies will be restricted to papers and abstracts published in the English language. For preclinical research studies, analyses must examine the associations between the bacterial gut microbiome and objective sleep measures or sleep disruption, restriction intervention, or circadian disruption intervention groups. For translational research protocols, analyses must contain the associations between the bacterial gut microbiome and objective or subjective sleep measures to compare across sleep pathology or intervention groups with the confirmation of sleep disruption (sleep inclusion groups defined in Table 1).

#### Exclusion Criteria

Review articles and studies that include the following are excluded: outcomes not related to the microbiome, analyses of the microbiome and sleep not performed, outcomes related to other gut microbiome organisms (virus, protozoa, and archaea, etc.), intervention used a symptom of sleep pathology but not alteration of sleep (ie, intermittent hypoxia), additional intervention not associated with the gut microbiome simultaneously used, other model organisms in preclinical research, and English language version not available.

### Search Strategy

Our research team met and developed relevant search terms to capture data sources covering the full scope of the intended aims, including the different iterations of sleep or sleep disruption, the gut microbiome, and human or rodent studies (for a full list of search terms and individual database search strategies, see Multimedia Appendix 1).

### Sources of Knowledge

We worked closely with our institutional medical research librarian to develop and refine our search strategy and Medical Subject Headings terms and identify pertinent databases to be searched. The search strategy and methods were peer reviewed by a medical librarian independent of the scoping review team to improve validity and reproducibility. The databases listed previously will be used to identify the primary sources of evidence. Secondary searches will be performed within Web of Science using snowballing technique conducting backward and forward citation analysis of the reference lists as well as the cited studies of the included studies for additional relevant articles.

The following databases will be searched:

- PubMed/MEDLINE (National Library of Medicine)
- Embase (Elsevier)
- Scopus (Elsevier)
- Web of Science: Core Collection (Clarivate Analytics)
- CENTRAL trials database (Wiley)
- BIOSIS Citation Index
- Zoological Record

No limitations will be placed on year of publication. Gray literature sources will also be searched, such as medRxiv and bioRxiv preprint servers.

### Study Records

#### Data Management

All information sources retrieved by the medical research librarian will be imported into Covidence systematic review screening software (Veritas Health Innovation), and duplicates will be removed. The data sources that pass initial screening for the full-text review will have PDF versions of the data source uploaded into Covidence by the medical research librarian. Naming conventions will be structured by the medical research librarian for the uploaded PDF files with an abbreviated title and year of publication. Excel spreadsheets will be used for the data collection of the included studies, with different tabs for preclinical and translational clinical studies. Naming and version control conventions using the date of last edit will be used for the data collection (synthesis table) Excel spreadsheet (eg, Sleep_Microbiome_Synthesis_Table_2022_04_24.xlsx). Synthesis tables will be stored in Box cloud-based storage system, and copies will be downloaded for backup regularly (at least weekly) on an institutional server. KAM will be responsible for managing the data resulting from this search and scoping review and after the project is completed.

#### Selection of Relevant Scientific Literature

The study screening and eligibility methods will be illustrated in a PRISMA (Preferred Reporting Items for Systematic Reviews and Meta-Analyses) flow diagram in the scoping review where the final number of included sources will be listed. Initially, 2 researchers (KAM and JA) will screen all the sources selected independently using the predefined inclusion and exclusion criteria in Covidence, and any discrepancies will be reviewed by a third researcher (GRW), where full agreement will be reached by all reviewers before a final decision is made to include a source. Prior to initiating, screening, and importing all sources into Covidence, a “pilot” of the protocol was performed by the 2 primary reviewing researchers where 15 randomly selected records were uploaded to ensure both reviewers had a shared understanding of the inclusion and exclusion criteria. The reviewers met regularly to discuss the consistency of their screening and data extraction form, revising the form if needed as additional microbiome outcome categories were identified and resolving disagreements through discussion.

#### Data Collection Process

After the final sources of evidence that will be included in the review are selected, data will be systematically collected using the synthesis table. Data charting will initially be performed by one team member (JA) and regularly audited by KAM (weekly) to ensure data abstracted from the included sources are consistent with the overarching research objectives and research questions. Data charting will be audited by other members of the research team as needed or if questions arise.

### Data Items and Outcomes

#### Synthesis Table

Key pieces of information from each study (author, year, aim of manuscript, and population) will be collected in a standard format on a separate tab on the synthesis table and presented in table format in the scoping review. Based on a research group discussion, we developed a priori preclinical sleep disruption categories (Table 1) and translational sleep disruption, subjective sleep metric, and objective sleep metric categories (Table 1). The included studies will be organized according to the type of sleep disruption model or the subjective or objective sleep assessment being studied, and preclinical and translational studies will be reported separately. The type of microbiome sequencing (ie, 16S ribosome ribonucleic acid amplicon sequencing or shotgun metagenomics sequencing) will be collected in the synthesis table. Microbiome, functional and metabolic gene, and metabolomics data will be collected, if reported, and annotated according to the synthesis table data columns outlined in Table 1. Cytokine and immune markers will also be extracted as both variables have known associations with sleep and the gut microbiome [74]. As we cannot assume directionality between sleep and microbiome metrics, we will focus on the associations between sleep pathology groups or measures and the gut microbiome instead of identifying specific outcome measures. Synthesis table data columns will be iteratively reviewed, and if it is determined by the study team that a key piece of information is not captured with the currently available extraction groups, an additional column will be added. If additional columns are added, previously reviewed studies will be re-examined to identify if new data need to be added to the synthesis table. Synthesis table data columns will be identical in the preclinical and translational clinical data collection tabs.

#### Risk of Bias in Individual Studies

One anticipated bias is the selective reporting of microbiome metrics across research studies reporting microbiome data. In an attempt to overcome this bias, we will collect all diversity (alpha and beta) metrics and other microbiome/metabolomics data related to our predefined data columns that are reported in the information source and supplemental data. We also will include data on the sample size, study population, and intervention used, if applicable, so that the synthesis of data can be examined in light of the populations or groups studied.

#### Synthesis of Results

The purpose of this scoping review will be to aggregate overarching findings and relationships with sleep quality and quantity with the gut microbiome, supplemented by functional gene, metabolite, and biologic marker associations, if available. Global microbiome, bacterial differential relative abundance, the predictive functional profiling of the gut microbiome community, and metabolomics results will be compiled in a standard format in both preclinical and translational studies. Individual bacterial taxa differential abundance between sleep disruption and sleep metric groups, along with bacterial relative abundance associations with sleep metrics, will be reported so agreement and disagreement across studies can be quantitatively analyzed. All data items and metrics aligning to the synthesis table data columns will be recorded in the synthesis table, but we will focus on metrics that have been reported across several studies to evaluate agreement or disagreement across the currently available data. The main results will be summarized, and the limitations of the scoping review process will be discussed. The collated data will be appraised for patterns and themes to identify possible pathways connecting sleep physiology and pathophysiology to gut microbiome functional and bacterial community characteristics. Possible physiologic pathways, the areas of study disagreement, and gaps in the literature will be presented to identify future areas of research and whether study replication is needed. We plan to use the PRISMA-ScR reporting guidelines to ensure all essential reporting items are included in the final scoping review [72].

## Results

The search strategy yielded 4622 references that were imported into Covidence systematic review screening software for study screening, and 87 duplicates were automatically identified and removed. Title and abstract screening of the 4535 remaining studies occurred, and full-text screening of the 154 full-text studies that passed title and abstract screening was completed in May 2022 by the 2 independent investigators identified in the methods section. A total of 93 sources were included for data extraction and synthesis. Data extraction of the identified sources will occur using the sleep and microbiome measures outlined in Textbox 1, and the synthesis table is expected to be completed by August 2022. The results of this scoping review will be disseminated through paper submission by December 2022 with the study findings and interpretation, along with presentation of the results to conferences related to neuroscience, sleep physiology, bioinformatics, and the microbiome.

## Discussion

Unreliable translation of preclinical studies to human populations and replication across human microbiome research studies has slowed progress in understanding the mechanisms and pathways underlying the connection of the microbiome to pathology and disease. Multiple factors contribute to bias in microbiome results, ranging from gut microbiome sample collection to sequence analysis [68,75,76], and the variability and heterogeneity in microbiome analysis metrics and statistical methods make standard cross-study comparison challenging. Therefore, we present our scoping review protocol in which we aim to extract, compile, and synthesize the primary sources of preclinical and translational clinical research focused on the relationships between sleep and sleep pathology with the gut microbiome. Our initial aim will be to survey which sleep disruption models have been used in preclinical research including mechanical sleep disruption [47], paradoxical sleep disruption [49], circadian light alteration sleep disruption [58], and biological sleep disruption [60] in gut microbiome research. We will also identify the extent that sleep disruption interventions have been used in human gut microbiome studies including sleep deprivation [63] and sleep restriction [51], and what pathological sleep conditions have been explored in the context of the microbiome. We hypothesize that sleep disruption will be associated with disruptions in global diversity measures and changes in specific bacterial taxa and metabolic genes, but these results will vary based on the microbiome analysis metric and sleep disruption model. Although several associations between sleep and gut microbiome community changes have been established in the currently available research, to our knowledge, this is the first systematic synthesis compiling specific bacterial and functional gut microbiome associations with sleep that takes the multiple sources of microbiome reporting bias into account. We hope this synthesis and presentation of results will inspire future microbiome researchers to comprehensively report and account for factors that potentially bias microbiome results [77]. Furthermore, we are working with other experts in the field to develop reporting standards and prioritize a consensus in base microbiome metrics to report across all research, regardless of statistical significance, to facilitate cross-study comparison and replication [78]. Our overarching aim of the proposed scoping review is to advance the current understanding of the mechanisms connecting the gut microbiome to sleep through gut-brain communication and the associated pathways. Therefore, we will initially synthesize and present the microbiome and related functional findings specific to each sleep measure or pathology, but we will also evaluate this data in relation to previously established pathways in the literature to provide researchers with opportunities to test the hypothesized mechanisms in future research. We will submit the findings of this scoping review for future publication to disseminate the results to the greater research community.

## Appendix

Multimedia Appendix 1 Base and database-specific search strategy.

## Abbreviations

PRISMA: Preferred Reporting Items for Systematic Reviews and Meta-Analyses
PRISMA-P: Preferred Reporting Items for Systematic Review and Meta-Analysis Protocols
PRISMA-ScR: Preferred Reporting Items for Systematic Reviews and Meta-Analyses Extension for Scoping Reviews
