# Supplementary material for: Examining the Relationships Between Sleep Physiology and the Gut Microbiome in Preclinical and Translational Research: Protocol for a Scoping Review
Source: JMIR Res Protoc. 2022 Jun 21;11(6):e38605. doi: 10.2196/38605 (PMC9257612; doi:10.2196/38605)
Supplement: Multimedia Appendix 1 [file resprot_v11i6e38605_app1.docx]

**Supplemental Table 1. Base and Database-Specific Search Strategy**

## **Supplemental Table 1:**

Database: PubMed
Provider: National Library of Medicine
Date Searched: 12/3/2021
Limits: English, editorial, commentary, retraction, reviews

|  | Concept | Search Strategy |
| --- | --- | --- |
| #1 | Microbiome | "Gastrointestinal Microbiome"[MeSH Terms] OR "metabolome"[MeSH Terms] OR "feces"[MeSH Terms] OR "intestines/microbiology"[MeSH Terms] OR "gut microbio*"[Title/Abstract] OR "gastrointestinal microbio*"[Title/Abstract] OR "fecal microbio*"[Title/Abstract] OR "microbiota"[Title/Abstract] OR "microbiome"[Title/Abstract] OR "16S"[Title/Abstract] OR "shotgun"[Title/Abstract] OR "metagenomics"[Title/Abstract] OR ("microbio*"[Title/Abstract] AND ("feces"[Title/Abstract] OR "fecal"[Title/Abstract])) |
| #2 | Sleep Disruption | "Circadian Clocks"[MeSH Terms] OR "circadian rhythm"[MeSH Terms] OR "sleep disorders, circadian rhythm"[MeSH Terms] OR "Sleep-Wake Transition Disorders"[MeSH Terms] OR "sleep disorders, intrinsic"[MeSH Terms] OR "Sleep Deprivation"[MeSH Terms] OR "Sleep Wake Disorders"[MeSH Terms] OR "sleep"[MeSH Terms] OR "circadian rhythm*"[Title/Abstract] OR "central clock*"[Title/Abstract] OR "sleep"[Title/Abstract] OR "sleeping"[Title/Abstract] OR "rapid eye movement*"[Title/Abstract] OR ("shift*"[Title/Abstract] AND ("work*"[Title/Abstract] OR "rotat*"[Title/Abstract] OR "evening"[Title/Abstract] OR "night*"[Title/Abstract])) |
|  | Limits | #1 AND #2 NOT (letter[ptyp] OR editorial[ptyp] OR comment[ptyp] OR news[ptyp] OR "Congress"[Publication Type] OR editorial[tiab] OR commentary[tiab] OR "Review"[Publication Type] OR "retracted publication"[ptyp] OR "retraction of publication"[ptyp] OR "retraction of publication"[tiab] OR "retraction notice"[ti] OR "retracted publication"[tiab] OR "Published Erratum"[Publication Type] OR corrigenda[tiab] OR corrigendum[tiab] OR errata[tiab] OR erratum[tiab]) Filters: **English** |
